# The forces driving cancer extracellular vesicle secretion

**DOI:** 10.1016/j.neo.2020.11.011

**Published:** 2020-12-14

**Authors:** Maarten P. Bebelman, Eline Janssen, D. Michiel Pegtel, Caitrin Crudden

**Affiliations:** 1 Department of Pathology, Cancer Center Amsterdam, Vrije Universiteit Medical Center, Amsterdam UMC, Amsterdam, The Netherlands; 2 Division of Medicinal Chemistry, Amsterdam Institute for Molecular Life Sciences, Vrije Universiteit, Amsterdam, The Netherlands

**Keywords:** Extracellular vesicles, Exosomes, Cancer cell signaling, Cancer therapy

## Abstract

The discovery that cancer cells discharge vast quantities of extracellular vesicles (EVs), underscored the explosion of the EV field. A large body of evidence now supports their onco-functionality in an array of contexts; stromal crosstalk, immune evasion, metastatic site priming, and drug resistance - justifying therapeutic intervention. The current bottleneck is a lack of clear understanding of why and how EV biogenesis ramps up in cancer cells, and hence where exactly avenues for intervention may reside. We know that EVs also play an array of physiological roles, therefore effective anticancer inhibition requires a target distinct enough from physiology to achieve efficacy. Taking the perspective that EV upregulation may be a consequence of the tumor landscape, we examine classic mutational events and tumor characteristics for EV regulators. All the while, aiming to illuminate topics worth further research in therapeutic development.

## Introduction

As with any multicellular entity, a tumor requires a complex network of cell-to-cell communication. Sustained growth, migration, multibarrier invasion, successful colonization and metastatic outgrowth requires bidirectional crosstalk within complex tissue environments. In addition to the secretion of soluble factors by cancer and/or stromal cells within the tumor microenvironment (TME), it has become clear that membrane encapsulated vesicles play a significant role. Packed with bioactive proteins, RNA and lipids, these extracellular vesicles (EVs; Box 1) are equipped to efficiently propagate oncogenic signals short and long distances. This mode of cellular crosstalk is not oncogenic *per se*, and occurs in many instances in normal organism development and homeostasis. However, its relevance in tumorigenesis has been particularly striking in the search for therapies. Hence, the cancer research community has been a core part of the exponential growth of the EV field. Cancer cells are commonly recognized to secrete much more EVs, and of altered composition, compared with their nonmalignant counterparts. Both the cancer originating-EVs, and also cancer-educated stromal originating-EVs, shape a tumor permissive environment. Such EVs have been implicated in many, if not all, steps of cancer progression including proliferation, angiogenesis, immune evasion, metastasis and drug resistance. The strength of the evidence is accumulating, and in vivo studies supporting EV-inhibition strategies in cancer control are growing rapidly. However, this work serves only as proof-of-concept for inhibiting the transfer of oncogenic EVs, as they all use strategies not feasible in patients.

The therapeutic potential is clear; what is not, is the target. We do not have a clear understanding of why and how EV biogenesis ramps up in cancer cells. This creates a research bottleneck, as a clear therapeutic window for exploitation is not apparent. Which signals drive EV biogenesis, what regulates oncogenic cargo decisions, what TME characteristics are significantly different from physiological EV-communication, that will allow intervention? Resolving the signals driving cancer EV biogenesis will fuel therapeutic development, yet a black box remains in place upstream. There are numerous excellent reviews on the many *roles* of cancer-derived EVs which we direct the reader to [6], [7], [8], [9], as we will not focus on this aspect. Instead, with this review we ask what *drives* tumor-specific EV communication, examine the current evidence for onco-specific regulators and speculate on what areas may represent vulnerabilities for therapeutic exploitation.

**Box 1 EV nomenclature**

EVs are a highly heterogeneous group of vesicles of different sizes, compositions and cellular origins [1]. The different EV subpopulations are often divided into two groups, based on the membrane they originate from; vesicles that directly bud from the plasma membrane are called microvesicles, while vesicles that bud in endosomal compartments and are secreted by exocytosis are called exosomes. Importantly, exosomes and microvesicles partially overlap in size, density and composition, making it impossible to distinguish these two groups from each other after isolating EVs from cell culture supernatant or biological fluids [1,2].Therefore, in line with the MISEV2018 guidelines from the International Society for Extracellular Vesicles, we will use the general term 'EV' when the cellular origin of the vesicle is unknown [3]. When size information is available, we will distinguish between small EVs (sEVs) that are smaller than 200 nm, and large EVs (lEVs) that are larger than 200 nm. We will use the terms microvesicles and exosomes only when the cellular origin of the vesicles follows from the biogenesis mechanism. For example, pyruvate kinase isozyme M2 (PKM2) phosphorylates the SNARE protein SNAP23, which mediates the fusion of multivesicular endosomes with the plasma membrane resulting in enhanced exosome secretion [4,5].

Alt-text: Unlabelled box

## Oncogenes and tumor suppressors governing EV secretion

Tumour-derived EVs support broad oncogenic processes such as angiogenesis [10], immune modulation [11,12], and metastasis [13], [14], [15], [16], [17]. Compared to non-malignant cells, cancer cells have been found to increase the amount of EVs produced [18], [19], [20], shift the subtype of EVs that they secrete [21], and/or alter the cargo molecules that are selected for expulsion [2,22,23]. Of note, education by tumor cells can also affect the secretion rate and the content of EVs derived from platelets [24], as well as noncancer cells in the TME [8], but in this review we chose to focus on EV secretion from cancer cells only. Theoretically, both changes in content and secretion rate could contribute to the oncogenic effects of tumor-derived EVs; increased secretion of EVs with unchanged composition could drive overstimulation of recipient cells, whereas changes in EV composition, by altering EV subtype or cargo loading, could bring about tumor-supportive microenvironmental remodeling. From an “identifying the regulators” perspective, control of either the secretion or composition of tumor EVs holds therapeutic potential. Either way, intervention aims to capitalize on unique differences between transformed and nontransformed cells in their EV production.

**Fig. 1:**
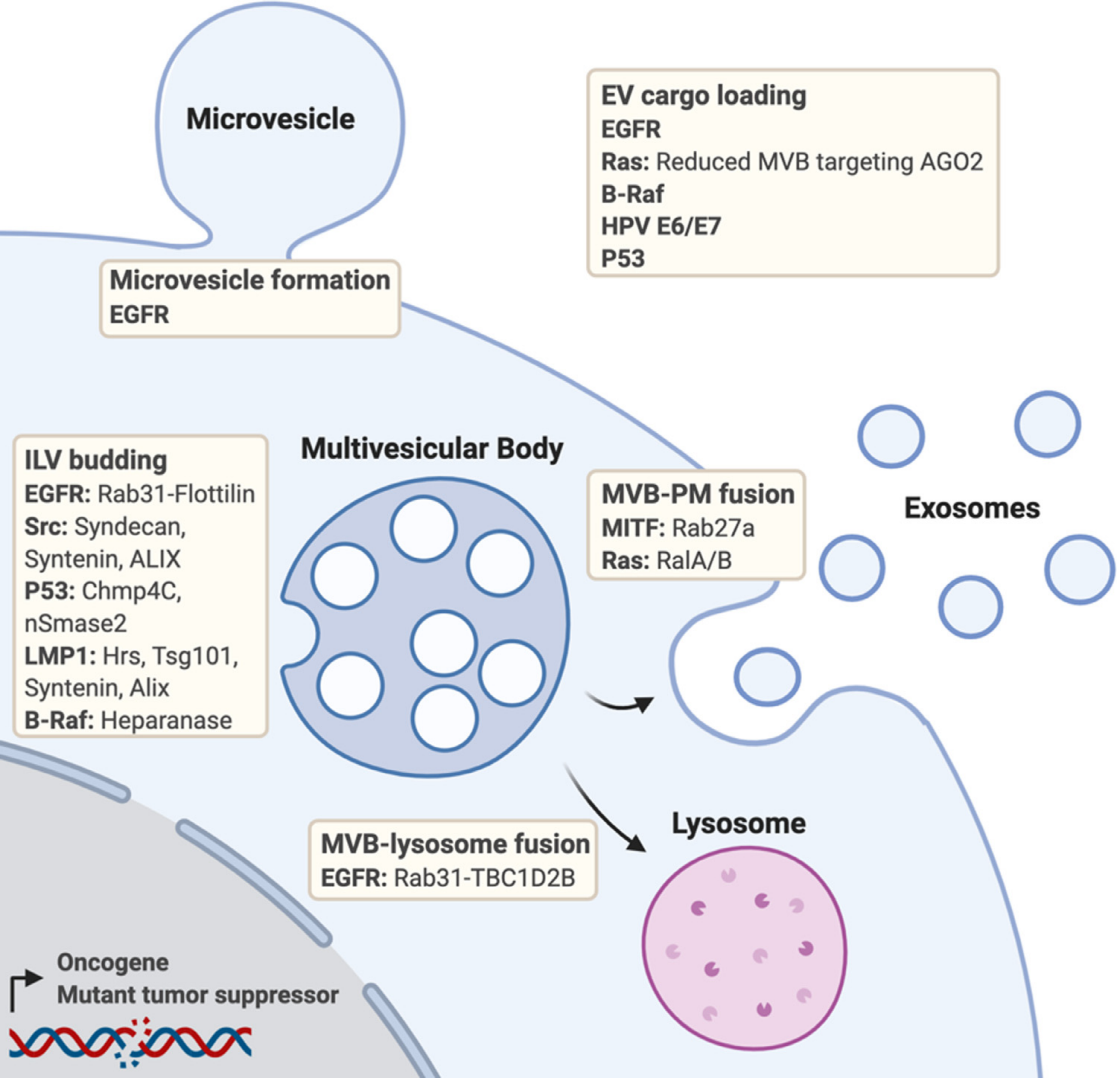
Overview of the roles of oncogenes and tumor suppressors at various stages of cancer extracellular vesicle secretion and cargo loading. Where known, the links between the oncogene/tumor suppressor and the extracellular vesicle biogenesis machinery is highlighted. Created with BioRender.com.

The activation of oncogenes from proto-oncogenes, as well as the inactivation or loss of tumor suppressor genes are key events in cancer progression. Oncogenes and tumor suppressors therefore have an intimate history with cancer research and target development, some with more success than others. Several oncogenes and tumor suppressors, either in their wildtype or mutated form, have been implicated in the regulation of EVs (Fig. 1). As classic “unique” genetic changes in cancer, they are interesting candidates for tumor-specific EV inhibition.

### EGFR

The epidermal growth factor receptor (EGFR) is a plasma membrane (PM) embedded receptor tyrosine kinase (RTK), that transduces a phosphorylation-driven signal cascade to promote cell survival and proliferation. EGFR hyperactivation occurs in many tumor types, through strategies such as receptor overexpression, autocrine stimulation, or constitutive active receptor mutants [25]. Therapies targeting either the receptor itself (blocking antibodies or tyrosine kinase inhibitors), or components of its downstream signal pathways are used clinically in many cancer types. Interestingly, the EGFR has been implicated in EV regulation in numerous contexts.

Expression of the constitutive active EGFRvIII mutant in the glioblastoma cell line U373 gives rise to an aggressive sub-line, with differential expression of multiple genes and proteins associated with EV biogenesis. U373-EGFRvIII show upregulation of certain tetraspanins linked to exosomes – TSPAN8, CD151, and downregulation of several other EV-related genes, including CD81 and CD82 [26]. The authors speculate that EGFRvIII reprograms the landscape of the cancer cell membrane, altering the vesiculation pathways. Aligning with this idea, EGFR signaling has been implicated in the formation of a subtype of very large (1-10 μm) membrane-derived microvesicles termed *large oncosomes* in prostate cancer cells [27]. Recently, constitutive active EGFR mutants have been found to drive their own secretion by promoting the production of a Rab31 dependent exosome subpopulation [28]. Mechanistically, active EGFR phosphorylates Rab31 on late endosomes, which in turn drives flotillin-dependent ILV formation. Simultaneously, active Rab35 inhibits the fusion of MVBs with lysosomes by recruiting the GTPase-activating protein (GAP) TBC1D2B that inactivates Rab7, thereby enhancing MVB exocytosis and exosome release.

By modulating EV subtype, EGFR activity can alter the global EV content expelled – mass spectrometry analysis in the glioblastoma study demonstrated that EVs released by EGFRvIII-transformed cells were enriched in focal adhesion related and pro-invasion proteins (CD44, BSG, CD151) [26]. Similar findings were reported for non–small cell lung carcinoma, where mutant EGFR-driven transformation changed the sEV proteome [23,29]. Interestingly, mutant EGFR itself can be secreted on EVs [26,29], and is linked to recipient cell proliferation [29], metastasis [30], and macrophage reprogramming [31]. Thus, in line with its well-characterized hyperactivity, EGFR-mutated tumor cells appear to carry alterations in their EV landscape, both in the subtype released and cargo selected.

### Oncogenic RAS mutations

Mutation and/or hyperactivation of Ras proteins (isoforms: N-Ras, H-Ras, K-Ras) drives around one-third of all human cancers. Ras proteins reside at the apex of core cellular proliferation/survival pathways, and therefore their dysfunction has delirious effects on shifting the balance towards uncontrolled growth. In addition, Ras activity also seems to regulate EV biogenesis rate. Oncogenic transformation of rat intestinal epithelial cells with mutant H-Ras increases the amount of EVs produced by these cells [32]. Moreover, Manumycin A, interfering with Ras farnesylation and thus activity, inhibits sEV biogenesis in castration-resistant prostate cancer cells, but not in non-transformed prostate epithelium cells [33]. A mechanistic link between Ras activity and EV release has yet to be elucidated. However, a recent study revealed a role for Rab13 in sEV secretion from colorectal cancer cells expressing mutant K-Ras [34]. Mutant Ras has also been reported to upregulate several proteins involved in ILV budding and MVB exocytosis [33]. Furthermore, the Ras-like GTPases RalA and RalB, which act downstream of Ras and contribute to Ras-induced tumorigenesis and metastasis [35,36], were found to control exosome secretion from mammary tumor cells [37].

Interestingly, multiple studies link Ras-mediated oncogenic transformation to changes in the microRNA (miRNA) and protein content of sEVs [38], [39], [40], [41]. Further research is necessary to see if the changes in EV protein content reflect changes in the cellular proteome, or are the result of altered EV cargo sorting in Ras mutated cells. In the case of miRNAs, mutant Ras does influence the sorting of specific miRNAs into exosomes [38]. Mechanistically, Ras-MEK signaling phosphorylates the RNA binding protein Argonaute-2 (Ago2), which inhibits its association with MVBs and consequently reduces the secretion of Ago2 and Ago2-bound miRNAs via exosomes [41].

Mutant Ras itself is enriched in sEVs from different Ras mutated cell lines, relative to the levels of wildtype Ras expelled by non-mutated counterparts [39,42,43]. Even though 2 post-translational modifications, farnesylation [44] and UBL3 modification [45], have been implicated in the sorting of Ras proteins to sEVs, neither is thought to regulate mutant Ras differently than wildtype. Therefore, it remains unclear what mechanistically drives the accumulation of mutant Ras in sEVs. The essentiality of farnesylation for the loading of K-Ras into sEVs in glioblastoma cells [44] does however, provide interesting indications for the use of farnesylation inhibitors to interfere with loading of (mutant) Ras.

### Additional human and viral oncogenes

In addition to EGFR and Ras, other well-known oncoproteins have been linked to cancer EV secretion and content. For example, 2 independent publications reported that the activation of Src promotes ILV budding and exosome secretion either via a direct interaction with ALIX [46], or via phosphorylation of syndecans and syntenin [47]. Microphthalmia-associated transcription factor (MITF) is an essential molecule for melanocyte proliferation and survival, which plays a key role in melanoma progression. MITF activity increases the expression of Rab27A [48], a small GTPase that controls the docking of MVBs to the PM [49], suggesting a role for MITF in release of melanoma exosomes. Mutant B-Raf signaling drives expression of heparanase, an enzyme previously described to enhance exosome secretion from breast cancer cells through a syndecan-syntenin-ALIX-dependent biogenesis route [50], [51], [52]. Finally, inhibition of mutant B-Raf changes the miRNA content of melanoma EVs [53]. Since the human genome contains over 300 oncogenes [54], it is likely that future research will reveal many more links between oncogenes and EV biogenesis. Besides these human oncogenes, oncoviruses such as Epstein-Barr virus (EBV) and human papillomavirus (HPV) have been found to express oncoproteins that affect EV biogenesis. The EBV latent membrane protein 1 enhances exosome production by increasing the expression of ILV budding machinery, including the ESCRT proteins Hrs and Tsg101, as well as Syntenin and ALIX [55]. Furthermore, both EBV and the related Kaposi's Sarcoma herpesvirus modulate the host sEV proteome [56]. In HPV-positive cervical cancer cells, expression of the HPV E6/E7 oncoproteins reduces the overall amounts of secreted EVs, but drives EV-mediated secretion of specific proteins and miRNAs [57], [58], [59].

### p53 loss and mutation

The tumor suppressor p53, which is activated upon cellular stress and controls decisions around cell cycle arrest, DNA repair and apoptosis, has been linked to EV release. Activation of p53 is reported to increase EV production [60], and 3 p53 target genes, *TSAP6* [60, 61], *Chmp4C* [62], and nSmase2 [63] have been implicated in this response in instances of radiation or DNA-damaging compounds. The involvement of the ESCRT protein Chmp4C and the ceramide producing enzyme nSmase2, both well-recognized for their role in intraluminal vesicle formation, suggest that p53 activation induces exosome release [62,63]. Thus, multiple studies report that wildtype p53 can control EV secretion dynamics in response to cellular stress. p53 is one of the most frequently mutated genes in human cancer, mainly involving mis-sense mutations that abrogate p53-mediated tumor suppression. Interestingly, loss of p53 results in increased sEV production from bone marrow mesenchymal stem cells, showing that depending on the cellular context both activation and loss of p53 function can enhance EV secretion [64]. To complicate matters even further, certain p53 mutations have been described that lead to novel protumorigenic activities of the protein. These so-called gain-of-function (GOF) mutations in p53 have been linked with changes in EV composition. In lung cancer cells, GOF-mutant p53 increases the release of sEV-associated extracellular Hsp90α, which promotes cancer cell invasion and metastasis [65]. Furthermore, in colorectal carcinoma cells, both loss of p53 and GOF-mutant p53 increase sorting of specific miRNAs into sEVs – both genotypes giving a different set of miRNAs. Among the enriched miRNAs, miR-1246 in sEVs derived from GOF-mutant p53 cells reprogram macrophages to be tumor supportive [66].

Combined, these studies show a highly context-dependent role for p53 in regulating EV secretion and composition. As with the oncogenes, the p53 pathway has been subject to intense therapeutic development, providing drug repurposing opportunities worth exploration for their impact on EV release in relevant scenarios.

## Tumor characteristics linked to EV upregulation

**Fig. 2:**
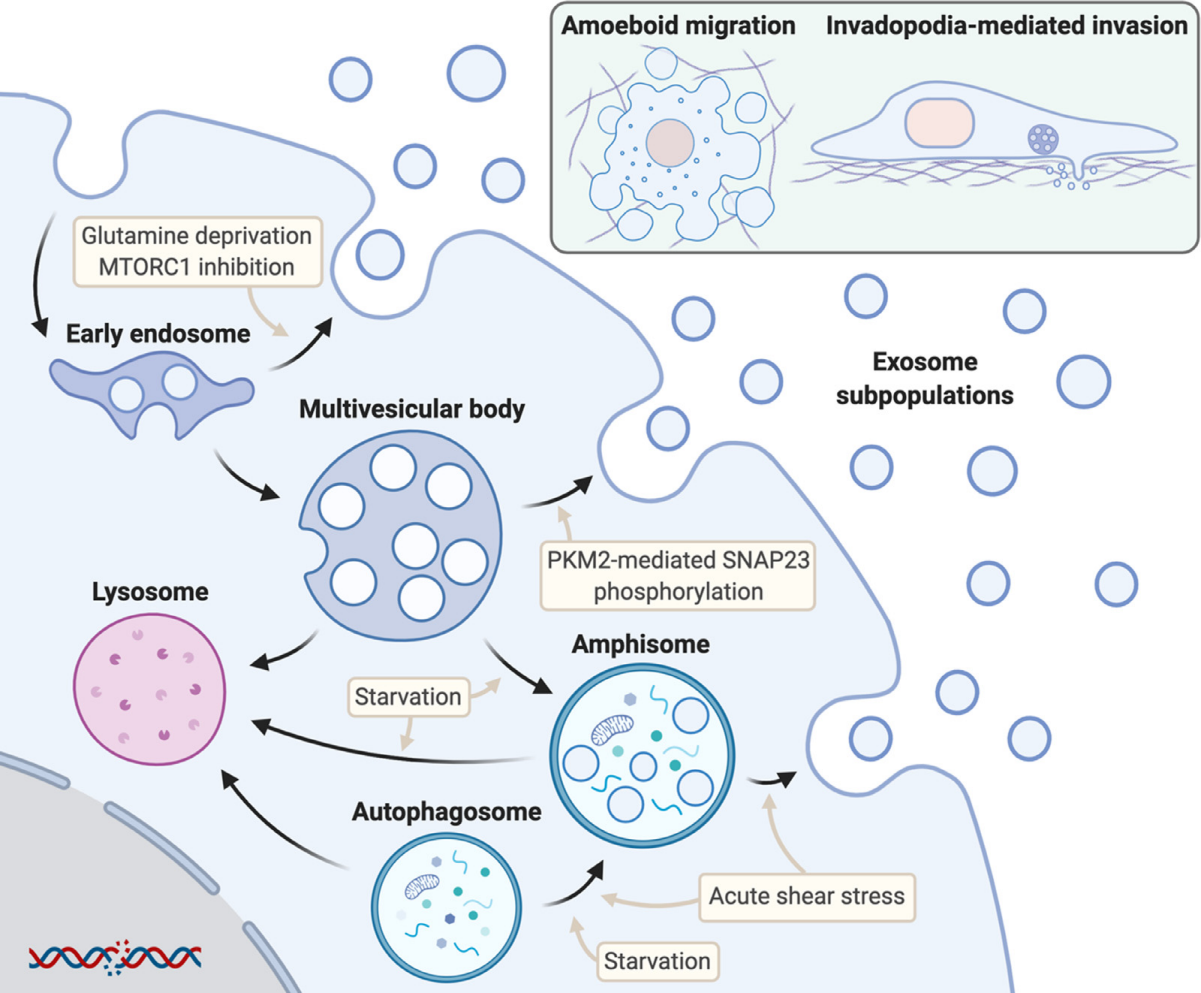
Overview of the influences of cell metabolism, autophagy and cell migration on cancer extracellular vesicle secretion. Cancer cells can release multiple subpopulations of exosomes from different compartments including early endosomes, multivesicular bodies and amphisomes. Upper right insert: Amoeboid migration and invadopodia-mediated invasion trigger the release of distinct extracellular vesicle subsets that help the migration of tumour cells through different types of extracellular matrix. Created with BioRender.com.

Despite the genetic heterogeneity of tumors inter- and intra-patient, there are some characteristics that are largely shared between cancer types. These include metabolic rewiring of cancer cells, alterations in autophagic flux and invasive behavior, all of which have been found to influence cancer EV secretion (Fig. 2).

### Metabolic reprogramming

Metabolic reprogramming has long been known to be a characteristic of many cancers, discovered by Otto Warburg in the 1920s. Despite oxygen availability, tumor cells often do not metabolize glucose via oxidative phosphorylation, but instead perform aerobic glycolysis. Even though this metabolic switch seems energetically unfavorable, it provides ample intermediate metabolites to fuel anabolic processes required for high rates of cell growth [67]. A key glycolytic enzyme is pyruvate kinase isozyme M2 (PKM2), which catalyses the final step of glycolysis – the conversion of phosphoenolpyruvate to pyruvate. PKM2 garnered particular attention after being shown as overexpressed and/or present in dimeric rather than tetrameric form in many tumor cells [68,69]. Tetrameric PKM2 is enzymatically active and facilitates energy production in tumor cells via aerobic glycolysis. Dimeric PKM2, on the other hand, carries out nonmetabolic processes – many that aid tumor progression [70], including directing intermediate carbon metabolites into synthetic processes [69]. It was recently shown that PKM2 also enhances cancer exosome release, linking metabolism to EV biogenesis [4]. Mechanistically, the dimeric form of PKM2 phosphorylates the exocytic SNARE protein SNAP23, which induces the fusion of MVBs with the PM. Besides this tumor intrinsic link between metabolism and EV secretion, extrinsic changes in cancer cell metabolism can also influence the production of cancer EVs. A recent publication reported that the depletion of glutamine from the TME causes a shift in the subtype of cancer EVs that are released; switching from late-endosomal CD63-positive exosomes towards RAB11a-positive exosomes that originate from recycling endosomes and promote tumor vascularization [21].

### Autophagy

Autophagy describes the delivery of cellular material to lysosomes for degradation, turning over cell components for energy and macromolecular precursors. As such, autophagy and exosome release could be viewed as 2 ends of the endolysosomal system. However, recent studies have described shared molecular machinery between exosome biogenesis and autophagy, as well as substantial crosstalk between these 2 processes [71]. In cancer, autophagy is often deregulated – but in sometimes opposing, context-dependent manners. In line with this, autophagy and components of the autophagy machinery have been described to both inhibit and contribute to EV secretion, depending on cancer type, cellular context, and the type of autophagy mechanism. Perhaps the clearest link between autophagy and exosome secretion is the fusion of MVBs with autophagosomes, leading to the formation of hybrid organelles termed amphisomes [72,73]. Early studies with the erythroleukemic cell line K562 showed that induction of autophagy by starvation or rapamycin treatment enhances the formation of amphisomes that subsequently fuse with lysosomes, resulting in decreased exosome release [74]. However, recent publications revealed that amphisomes can also fuse with the PM, leading to the secretion of exosomes as well as a subtype of small EVs that contains autophagy-related cargo [75], [76], [77]. The secretion of EVs from amphisomes is especially increased when the fusion of MVBs and autophagosomes with lysosomes is blocked [76]. In addition, metastasizing tumor cells that travel through the blood or lymphatic vessels experience acute shear stress that promotes autophagy. Both amphisome formation and the secretion of autophagy components in amphisome-derived EVs is highly elevated in these cells, suggesting a role for amphisome-dependent EV secretion in the maintenance of cellular homeostasis in metastasizing cancer cells [77].

The mechanistic target of rapamycin complex 1 (mTORC1) is a key regulator of autophagy, regulating the balance of cell growth and autophagy based on nutrient availability, growth factor signaling and cellular stress. In cancer, mTORC1 signaling is often enhanced, leading to inhibition of autophagy and stimulation of cell growth [78]. Various studies have linked mTORC1 activity to changes exosome secretion, but again the direction of these changes varies per cell type. In hepatic stellate cells mTORC1 signaling enhances EV secretion [79], while in mouse embryonic fibroblasts mTORC1 signaling inhibits Rab27a-dependent exosome secretion [80]. In various cancer cell lines, mTORC1 inhibition with rapamycin or via glutamine deprivation induce the secretion of protumorigenic RAB11-positive exosomes [21].

Besides classical autophagy, various autophagy related genes (ATGs) have been found to contribute to EV secretion independent from their role in autophagy. For example, ATG12 and ATG3 control ALIX-mediated exosome biogenesis [81], and ATG7 drives LC3-dependent cargo recruitment into ILVs [82]. Furthermore, ATG5 mediates the dissociation of the vacuolar proton pump (V_1_V_0_-ATPase), which prevents acidification of the MVB lumen and results in increased MVB-PM fusion and exosome release [83].

In conclusion, there is sufficient evidence that autophagy and ATGs are tightly connected to cancer EV secretion. However, differences in cancer type, nutritional status and TME are likely to affect the consequences of autophagy on cancer EV secretion. Further research is required to determine under which circumstances therapeutic manipulation of autophagy could be used to inhibit the production of cancer EVs.

### Cell migration and invasion

The migration and invasion of cancer cells into surrounding tissue and vasculature is an important initial step in metastasis. A body of work from the D'Souza-Schorey group describes a scenario whereby the motility process itself may regulate microvesicle release from cancer cells. During cell invasion through deformable environments, tumor cells adopt an amoeboid phenotype – described as “rounded and high blebbing morphology” and release microvesicles, not evident in firm matrices wherein cells switch to a flat and elongated phenotype and generate invadopodia [84]. Mechanistically, the small GTPases ARF6 and RhoA regulate microvesicle secretion by controlling myosin light chain phosphorylation [84,85]. Blebbing microvesicles contain the metalloproteinase MT1-MMP and have the ability to degrade extracellular matrix, suggesting a role for these vesicles during amoeboid cell migration [85,86]. On the other hand, invadopodia have been found to act as docking sites for MVBs, and invadopodia formation enhances exosome secretion [87]. Similar to the blebbing microvesicles, these exosomes contain metalloproteinases and contribute to matrix degradation and stabilization of nascent invadopodia. Combined, these studies show that depending on their environment migrating cancer cells can secrete specific EV populations that help them invade tissues.

## Tumor microenvironmental impact on EV biogenesis

Cell-autonomous factors, such as those discussed above, are not the sole drivers of cancer progression, nor of tumor-specific exosome biogenesis. A “successful” cancer requires cancer/stromal cell co-evolution, giving rise to the state of the TME. Examples of EV-mediated stromal “education” are numerous – but the origins lie again in the tumor cell – reorienting our search for the *drivers* back to the same place. However, the particular make-up of solid tumors perpetuates certain TME characteristics aside from cells, worth exploration.

### pH

As a consequence of their metabolic reprogramming, tumor cells acidify the TME by secreting high concentrations of lactic acid. Once considered a mere waste product, it is now clear that lactate serves as an important onco-metabolite, and TME acidosis serves several roles, including governing an immunosuppressive environment [88,89]. Interestingly, various cancer cell lines from different tumor types secrete increased amounts of EVs when cultured under acidic conditions [90], [91], [92]. In the case of (intermediate stage) melanoma cells, low pH culture medium changes the lipid [90] and protein composition [91] of the secreted EVs. The significance of such changes is illustrated by the ability of these EVs to increase the invasiveness and migratory capacity of melanoma cells cultured under pH neutral conditions [91]. EV biology is deeply entrenched into membrane function, so it is also important to consider what effects acidification has on membrane biophysical properties. In acidic conditions, the membrane rigidity and lipid composition of those melanoma secreted vesicles were shown to be changed - high rigidly and sphingomyelin/ganglioside GM3 content [90]. The authors speculate on whether these components are responsible for the increased fusion activity. More broadly, oncogenic changes to membrane integrity and how they impact EV biogenesis is an interesting perspective.

### Hypoxia

Hypoxia, defined as insufficient oxygen to carry out efficient cellular metabolism, often occurs in solid tumors as they outgrow their vascular supply. Hypoxia is considered to be an element of the TME that can regulate tumor aggressiveness by inducing transcriptional reprogramming [93]. In part, hypoxia might promote tumor aggressiveness by assigning protumorigenic capacities to cancer-derived EVs. Hypoxic conditions increase the amount of EVs produced by various tumor cells [94], [95], [96], [97] and change the EV protein [98,99] as well as miRNA [17,[94], [95], [96],[100], [101], [102]] profile in different tumor types. As a result, hypoxic tumor EVs obtain characteristics that enable them to stimulate angiogenesis [95,96,98,100], tissue invasion, metastasis [17,99], and immune modulation [102,103] by altering the behavior of recipient cells.

Despite multiple lines of evidence suggesting that hypoxia drives cancer EV biogenesis, the molecular details are poorly understood. Not surprisingly, the hypoxia-induced transcription factors HIF-1 and HIF-2 were found to mediate hypoxia-induced EV release in breast cancer cells [94,104], suggesting that HIFs promote expression of EV biogenesis machinery [4,105]. PKM2, for example, is such a HIF1 target gene with a role in exosome secretion. Furthermore, HIF-induced expression of Rab22A promotes microvesicle budding under hypoxic conditions in various breast cancer cell lines [104]. In addition to HIF1, phosphorylation of proline-rich Akt substrate of 40 kDa (PRAS40) as well as activation of STAT3 have been found to contribute to hypoxia-induced cancer EV release, the latter most likely by influencing Rab7 and Rab27A expression levels [97,106].

Taken together, a hypoxic TME seems to drive tumor-specific EV biogenesis by stimulating various incompletely understood signaling pathways. Even though hypoxia itself might not be easily targetable, interfering with hypoxia-induced deregulated pathways might be an attractive EV inhibition strategy. Altogether indicating that the TME should not be overlooked in the search for therapies targeting tumor-specific EV biogenesis.

### Immune cell interactions

Avoidance of immune detection is necessary for a cancer to progress past a certain stage, and hence a feedback relationship between immune cells and cancer cells is a pan-disease characteristic. There is some evidence to suggest that EV release can be reactive to immune signals.

From a biogenesis standpoint, an immune regulation pathway links to MVB biogenesis. ISG15 is an interferon (IFN)-α/β-inducted UBL that can be conjugated to target proteins, termed ISGylation. The procedure is similar to ubiquitin action, but occurs predominantly in virus-infected cells, interfering with virus assembly and function. ISG15 expression can block virus-budding via mechanism such as blockage of ESCRT machinery in virus-infected cells. Exosomes too, utilize the ESCRT machinery. The Sanchez-Madrid group discovered that ISGylation of the MVB protein Tsg101 initiates degradation, re-routing MVBs towards lysosomal destruction instead of exosome release [107]. ISGylation induced by either ISG15 machinery overexpression or IFN-I is sufficient to decrease exosome release, with no impact on microvesicles. In a noncancer context, chemokines – molecular messengers of the immune system – have been shown to be linked to EV release. In hepatocytes the release of exosomes is dependent upon chemokine receptors CXCR1 and CXCR2, in a mechanism through neutral sphingomyelinase and intracellular ceramide [108]. CXCR1-deficient hepatocytes produced fewer exosomes, whereas CXCR2-deficient hepatocytes produced more exosomes than wild-type controls. There are also examples of EV cargo specificity regulated by immune signals. The programmed-death receptor 1/ligand interaction governs “checkpoint inhibition,” to signal when it is not appropriate for an immune response. PD-L1 is released on EVs from many cancer cells, in response to interferon-γ (IFN-γ) stimulation [109], in a physiological context provided by T cells.

Cancer EV expulsion can be driven by the cells encountering different situations, including immune stimuli. In the current era, where immunotherapy is making its way into oncology regimes, understanding what EVs contribute to immune-cancer crosstalk needs to be better understood, to truly tap into the therapeutic potential of intervention.

## Concluding remarks

The process of vesiculation is distorted during malignant transformation, and hence altered EV handling appears to be a pan-cancer characteristic. Changes take several forms – EV emission rates, size, subtype, molecular composition, and biological activity. This review considers the possibility that classic “hallmarks” such as altered signal cascades, or microenvironmental status cause a shift toward EV release. Preliminary research certainly suggests that there may be distinct cascades or processes upstream of oncogenic-EV mediated communication, which may carry targetable components.

At one stage, the EV field had many opponents who maintained an artefact perspective over true functionality. However, it is now difficult to argue with the evidence of their tumor-supportive roles in a host of in vivo models, and the clear benefit of inhibition in certain contexts. The field is now primed to leverage this position, and needs targets for therapeutic intervention. Herein we review aspects of the tumor cell proposed to be linked to EV biogenesis and/or cargo decisions, worth exploration. As many of these aspects have themselves been under therapeutic scrutiny for some time, the EV field has a host of options to explore in drug repurposing studies. The more pieces we can add to the molecular mechanism puzzle of EV biogenesis, the closer to get to shifting EV inhibition from proof-of-concept to clinical feasibility.

## Authors Contribution

M.P. Bebelman: Data Curation, Writing - Original draft preparation, Writing - Review & Editing, Visualization. E. Janssen: Data Curation, Writing - Original draft preparation. D.M. Pegtel: Conceptualization, Writing - Reviewing and Editing, Supervision. C. Crudden: Conceptualization, Data Curation, Writing - Original draft preparation, Writing - Review & Editing, Supervision.

## Conflicts of Interest

The authors declare the following financial interests/personal relationships which may be considered as potential competing interests: D.M. Pegtel is a cofounder and CSO of ExBiome BV as well as an advisor to Takeda. This work was supported by grants to D.M. Pegtel by the Dutch Cancer Society (KWF Unique High Risk Project 2017-2; 11308), an NWO (AIMMS STAR Graduate Program grant 022.005.031) to M.P. Bebelman, and a Marie Sklodowska-Curie Fellowship from the European Commission (H2020-MSCA-IF-2018; 845391) to C. Crudden.
